# GC-MS Analysis: *In Vivo* Hepatoprotective and Antioxidant Activities of the Essential Oil of *Achillea biebersteinii* Afan. Growing in Saudi Arabia

**DOI:** 10.1155/2016/1867048

**Published:** 2016-05-12

**Authors:** Mansour S. Al-Said, Ramzi A. Mothana, Mohammed M. Al-Yahya, Syed Rafatullah, Mohammed O. Al-Sohaibani, Jamal M. Khaled, Abdulrahman Alatar, Naiyf S. Alharbi, Mine Kurkcuoglu, Husnu C. Baser

**Affiliations:** ^1^Department of Pharmacognosy, and Medicinal, Aromatic & Poisonous Plants Research Center (MAPPRC), College of Pharmacy, King Saud University, P.O. Box 2457, Riyadh 11451, Saudi Arabia; ^2^Department of Pathology, King Khalid University Hospital, King Saud University, P.O. Box 2925, Riyadh 11451, Saudi Arabia; ^3^Departments of Botany and Microbiology, College of Science, King Saud University, Riyadh 11451, Saudi Arabia; ^4^Department of Pharmacognosy, Faculty of Pharmacy, Anadolu University, 26470 Eskisehir, Turkey; ^5^Department of Pharmacognosy, Faculty of Pharmacy, Near East University, Nicosia, Northern Cyprus, Mersin 10, Turkey

## Abstract

Liver disease is a worldwide problem. It represents one of the main causes of morbidity and mortality in humans.* Achillea biebersteinii* is used as herbal remedy for various ailments including liver diseases. But the scientific basis for its medicinal use remains unknown. Thus, this research was undertaken to evaluate the efficiency of* A. biebersteinii* essential oil (ABEO) (0.2 mL/kg) in the amelioration of CCl_4_-induced hepatotoxicity in rodent model. Moreover, the chemical content of the oil was investigated using GC and GC-MS. The following biochemical parameters were evaluated: serum glutamic oxaloacetic transaminase (GOT), glutamic-pyruvic transaminase (GPT), gamma-glutamyl-transpeptidase (*γ*-GGT), alkaline phosphatase (ALP), and total bilirubin. Furthermore, lipid profile, malondialdehyde (MDA), nonprotein sulfhydryl (NP-SH), and total protein (TP) contents in liver tissue were estimated. 44 components (92.0%) of the total oil have been identified by GC-MS analysis where *α*-terpinene and *p*-cymene were the most abundant. The high serum enzymatic (GOT, GPT, GGT, and ALP) and bilirubin concentrations as well as the level of MDA, NP-SH, and TP contents in liver tissues were significantly reinstated towards normalization by the ABEO. Histopathological study further confirmed these findings. In addition, ABEO showed mild antioxidant activity in 2,2-diphenyl-1-picrylhydrazyl (DPPH) radical scavenging and *β*-carotene-linoleic acid assays.

## 1. Introduction

In human, liver disease which is a worldwide problem is considered one of the most important diseases that cause a significant proportion of morbidity and mortality in all age-groups. World health organization (WHO) estimated that chronic hepatitis c patients reached approximately 170 million people worldwide and 3-4 million people are annually added to that list. In addition, it is reported that hepatitis B virus (HBV) causes annually more than 2 billion infections and over 5 million people are being affected annually. The available treatment options for liver diseases are predominantly limited in efficiency, have often serious side effects, and are too costly especially for the developing country [1–3]. The key features of therapeutic agent are a high effectiveness and low threat of side effects. Thus, improvement of novel and more substantial hepatoprotective drugs with minor side effects is obligatory. Many medicinal herbs are showing such characteristics and might represent new active therapeutic drugs.

The genus* Achillea* (family: Asteraceae) is represented by four species in Saudi Arabia.* Achillea biebersteinii* Afan., locally known as “Althufra,” is native to the Arabian Peninsula, including Saudi Arabia, Yemen, and Oman, as well as to countries around the Mediterranean Sea.* A. biebersteinii* is a perennial herb, 30 to 60 cm high. The aerial part of the plant is commonly used in the form of decoction by folkloric practitioners for the treatment of several ailments including abdominal pain, wound healing, jaundice, and other liver diseases. In phytochemical and pharmacological studies, the antibacterial, antifungal, and antioxidant activities of the plant have been reported [4–6]. To the best of our knowledge, no previous investigation on the protective activity of the essential oil of* A. biebersteinii* against CCl_4_-induced hepatotoxicity in rats has been reported. Thus, the current study was undertaken to evaluate the protective effect of the essential oil on CCl_4_-induced hepatotoxicity and to elucidate the possible mechanisms underlying these protective effects in rats. Moreover,* in vitro* antioxidant activities were assessed by DPPH radical scavenging and *β*-carotene linoleic acid assays.

## 2. Materials and Methods

### 2.1. Plant Material

The herb of* Achillea biebersteinii* was collected from south of Saudi Arabia in February 2010. The identification and authentication of the plant were performed by Dr. Mohammed Yousuf and deposited (voucher specimen (# 5610)) at the Herbarium of the College of Pharmacy, King Saud University, Riyadh, Saudi Arabia.

### 2.2. Extraction of the Essential Oil

The aerial parts of* A*.* biebersteinii* were dried and powdered and then extracted by hydrodistillation method for 3 h according to the European Pharmacopoeia using a Clevenger-type apparatus. Anhydrous sodium sulfate was used to dry the obtained oil (ABEO) and after that the oil was filtered and stored at +4°C until the following analysis and tests.

### 2.3. Gas Chromatography Analysis

An Agilent 6890N GC system was used to perform the GC analysis. A temperature of FID detector was adjusted to 300°C and identical operational settings used in a duplicate of the same column were applied in GC-MS analysis. Synchronous autoinjection was carried out to achieve the same retention times. Relative proportion amounts of the separated compounds were counted from integration of the peaks in FID chromatogram.

### 2.4. Gas Chromatography-Mass Spectrometry

The GC-MS analysis was performed with an Agilent 5975 GC-MSD system with Innowax FSC column (60 m × 0.25 mm, 0.25 *μ*m film thickness). Helium was used as carrier gas at a flow rate of 0.8 mL/min. Oven temperature was programmed to 60°C for 10 min and raised to 220°C at rate of 4°C/min. Temperature was stilled constant at 220°C for 10 min and then upraised to 240°C at a rate of 1°C/min. Mass spectra were recorded at 70 eV with the mass range *m*/*z* 35 to 450.

### 2.5. Identification of Compounds

The identification of ABEO components was done by comparing the retention times of oil components with standard samples or by comparing the relative retention indices to series of* n*-alkanes. Computer matching against commercial (Wiley GC/MS Library, Adams Library, and MassFinder 2.1 Library) [7, 8] and in-house library, “Baser Library of Essential Oil Constituents,” built up by genuine compounds and components of known oils, as well as MS literature data [9, 10], was used to identify the essential oil components.

### 2.6. Animals

Wistar albino rats of either sex were procured from Experimental Animal Care Center, College of Pharmacy, King Saud University, Riyadh. Rats were approximately 8–10 weeks old and weighed 180–200 g. For the sleeping time and acute toxicity tests, Swiss albino mice were used. The animals were kept in controlled environment at 23 ± 2°C (temperature), 55% humidity, and 12 h light : 12 h dark cycle. Drinking water and Purina chow diet* ad libitum* were provided. The procedure of the present study was permitted by the Ethics Committee of the Experimental Animal Care Society, College of Pharmacy, King Saud University, Riyadh.

### 2.7. Experimental Design

#### 2.7.1. Acute Toxicity Test

To evaluate the acute toxicity of* A*.* biebersteinii* essential oil on mice, several doses were tried. The different groups were administered with numerous doses (0.1–0.5 mL/kg) using the oral route. The clinical signs and symptoms of toxicity were observed continuously after treatment for 4 h (1, 1:30, 2, 2:30, 3, 3:30, and 4 h) and then after 72 h. The mortality was recorded daily for 14 days [11].

#### 2.7.2. Carbon Tetrachloride-Induced Liver Toxicity

The rats were randomly divided into 4 groups (6 rats per group); after that each group randomly was named (control, CCl_4_ only, CCl_4_ + ABEO, and CCl_4_ + silymarin). The control group was kept without treatment while other groups were administrated intraperitoneally (IP) with 1.25 mL/kg of CCl_4_ body weight. CCl_4_ + ABEO and CCl_4_ + silymarin group were given 0.2 mL/kg of* A*.* biebersteinii* essential oil (ABEO) and silymarin at a dose of 10 mg/kg orally. The treatment by ABEO and silymarin was started 3 weeks prior to CCl_4_ administration and continued until the end of the experiment. 24 hours after the CCl_4_ treatment, the blood was collected from all groups and then the serum was separated from clotted blood. Ether anesthesia was used to sacrifice the animal after collecting the blood. The liver was dissected to perform the biochemical and histological examination.

#### 2.7.3. Estimation of Marker Enzymes and Bilirubin

Serum glutamate oxaloacetate transaminase (SGOT), serum glutamate pyruvate transaminase (SGPT), alkaline phosphatase (ALP), gamma-glutamyl transferase (GGT), hemoglobin, and bilirubin were determined using a Reflotron® Plus Analyzer and Roche kits [12–15].

#### 2.7.4. Estimation of the Lipid Profile

Commercial diagnostic kits were used to estimate total cholesterol, triglycerides, high-density lipoproteins (HDL-C), and glucose levels [16–18].

#### 2.7.5. Determination of Malondialdehyde (MDA)

The protocol explained by Utley et al. [19] was followed. Potter-Elvehjem type C homogenizer was used to homogenize the liver tissues in KCL solution (0.15 M) at 4°C to obtain 10% of tissue (w/v). Metabolic shaker was used to incubate 1 mL of the homogenate at 37°C for 3 h. Then the homogenate was mixed with 10% aqueous trichloroacetic acid (1 : 1). The mixture was separated into supernatant and sediment by centrifugation at 800 ×g for 10 min. 1 mL of thiobarbituric acid (0.67% in water) was add to 1 mL of the supernatant and then was placed in water bath at 100°C for 10 min. After cooling, the dilution was done by 1 mL distilled water. The optical density of the solution was recorded at 535 nm by spectrophotometer and then the concentration of malondialdehyde (nmol/g wet tissue) was calculated from a standard curve of malondialdehyde solution.

#### 2.7.6. Estimation of Nonprotein Sulfhydryls (NP-SH)

Hepatic nonprotein sulfhydryls were estimated according to the assay of Sedlak and Lindsay [20]. Homogenization of liver tissue samples was performed in ice-cold ethylenediaminetetraacetic acid (EDTA) (0.02 mmol/L). 1 mL of 50% trichloroacetic acid (TCA) and 4 mL distilled water was added to 5 mL of the homogenate placed in 15 mL test tube. After shaking for 10 min, the tubes were centrifuged at 3000 rpm and then 2 mL of supernatant was removed and mixed with 4 mL Tris buffer (0.4 mol/L, pH 8.9). The mixture was shaken with 0.1 mL of 5,5′-dithio-bis (2-nitrobenzoic acid) (DTNB) for 5 min; then immediately the optical density at 412 nm was measured by spectrophotometer against a reagent blank.

#### 2.7.7. Determination of Total Protein (TP)

Total protein in serum (g/L) was determined by colorimetric method using total protein kit CS610 (Crescent Diagnostics, Jeddah, Saudi Arabia). The colorimetric assay for determining total protein concentration in serum is based on the chemical reaction between copper ion (in biuret reagent) and peptide bonds (in serum proteins) in alkaline solution to give a blue/violet color. Tartrate and iodide are added as stabilizer and inhibitor of autoreduction of the alkaline copper complex. The optical density of the blue/violet colored complex was estimated at 546 nm by short-cut-assay. The serum total protein was calculated using the following equation:

(1) Total protein in serumg/L=AbssampleAbsstandard×concentration of standard,

where the Abs_sample_ mean absorbance of sample and Abs_standard_ refer to absorbance of standard protein (g/L).

#### 2.7.8. Histopathological Assessment

The microsection of liver tissues (5 *μ*m thickness) was produced by American-made optical rotary microtome according to VIP tissue processor. The pathomorphological changes in the microsection of liver tissues stained with hematoxylin and eosin stain were studied by light microscope [21].

### 2.8. Studies of Antioxidant Activity

#### 2.8.1. Scavenging Activity of DPPH Radical

The antioxidant activity of ABEO was evaluated by DPPH free radical scavenging assay according to Brand-Williams et al. [22]. In the presence of an antioxidant, the DPPH radicals (purple color) turn to reduced DPPH (yellow color). The change of the optical density is recorded by spectrophotometer at 517 nm. Five concentrations 10, 50, 100, 500, and 1000 *μ*g/mL of ABEO were prepared. Briefly, the total volume of test mixture containing 500 *μ*L of ABEO, 125 *μ*L DPPH, and 375 *μ*L solvent was mixed and then incubated at 25°C for 30 min. The change in color was determined at 517 nm by spectrophotometer (UVmini-1240, Shimadzu, Japan). As positive control in this assay, the known antioxidant ascorbic acid was used. The radical scavenging activity was measured using the following equation:

(2) Radical scavenging activity%=Abscontrol−AbssampleAbscontrol×100.

#### 2.8.2. *β*-Carotene-Linoleic Acid Assay

To determine the antioxidant activities of ABEO, the *β*-carotene bleaching assay was performed according to Mohd-Esa et al. [23]. *β*-carotene solution (0.2 mg/mL) was prepared with chloroform; then 1 mL of this solution was added to a solution of 0.2 mL of Tween-20 and 0.02 mL of linoleic acid. Using rotary evaporator the chloroform was removed. The mixture was diluted with 100 mL of distilled water and mixed for 2 min. A 5 mL of the mixture was mixed with 0.2 mL of ABEO (1 mg/mL) and incubated in a water bath at 40°C for 2 h. The optical density was recorded at 470 nm at 15 min intervals, by a UV-visible spectrophotometer (UVmini-1240, Shimadzu, Japan). Rutin was used as a positive control prepared (1 mg/mL). The antioxidant activity was estimated using the following equation:

(3) $$\begin{array}{c} \text{Antioxidant activity}\left(\;\%\;\right)=1-\frac{\left({Abs}_{0}-{Abs}_{t}\right)}{\left({Abs}_{0}^{{}^{\circ}}-{Abs}_{t}^{{}^{\circ}}\right)}\times 100, \end{array}$$

where Abs_0_ and Abs_0_° are the optical density values determined at zero time of incubation for ABEO and control, respectively. Abs_*t*_ and Abs_*t*_° are the optical density values for ABEO and control, respectively, at 120 min.

### 2.9. Statistical Analysis

A completed random design (CRD) was applied in the present study. The results are expressed as mean ± standard error. The data were statistically analyzed by ANOVA, followed by Dunnett's multiple comparison test.

## 3. Results

### 3.1. Composition of the Essential Oil

Hydrodistillation of the aerial parts of* Achillea biebersteinii* afforded yellow oil with a yield of 0.70% (w/w) on dry weight basis. The Kovats retention indices, percentage composition, and identification tools are demonstrated in Table 1. The identified compounds are arranged according to their elution on the Innowax FSC column. 44 components (92.0%) of the total oil of* A*.* biebersteinii* (ABEO) have been identified by GC-MS analysis. According to the literature review, nothing was available concerning the qualitative and quantitative analysis of the volatile oil of* A*.* biebersteinii* growing in Saudi Arabia. The data in Table 1 indicated that the monoterpene hydrocarbons content of ABEO was high (56.3%). The monoterpene hydrocarbons alfa-terpinene and* p*-cymene were the most abundant compounds and represented 29.2 and 22.9%, respectively. Moreover, oxygenated monoterpenes accounted for 31.9% of the total oil, with terpinen-4-ol (4.7%), 1,8-cineole (4.3%),* trans*-*p*-menth-2-en-1-ol (3.9%), ascaridole (3.1%),* trans*-piperitone oxide (2.5%), and carvacrol (2.1%) as the major compounds.

### 3.2. Acute Toxicity

The* A*.* biebersteinii* essential oil (ABEO) at various dose concentrations did not demonstrate any mortality or any pathological symptoms even at the highest dose (0.5 mL/kg, p.o.). Based on that, one dose (0.2 mL/kg) was chosen for further pharmacological investigations.

### 3.3. Effect of ABEO on Marker Enzymes and Bilirubin in Serum

The results of the biochemical indicators of liver function are summarized in Table 2. Administration of CCl_4_ significantly caused severe hepatotoxicity in rats, as evidenced by the elevation of serum GOT, GPT, ALP, GGT, and bilirubin. The elevation of the enzymes and bilirubin in serum was significantly diminished in pretreated rat groups with ABEO (0.2 mL/kg) as well as with silymarin (10 mg/kg) when compared with the CCl_4_ only treated group.

### 3.4. Effect of ABEO on Lipid Profile

As demonstrated in Table 3, CCl_4_ showed a significant increase in the level of lipid profile in rats. Administration of ABEO attenuated significantly the increased level of cholesterol and triglycerides in rats treated by CCl_4_ compared to the normal control group. Moreover, silymarin as well showed a significant reduction of lipid profile in rats compared to those of group treated only by CCl_4_.

### 3.5. Effect of ABEO on Hepatic MDA

**Figure 1:**
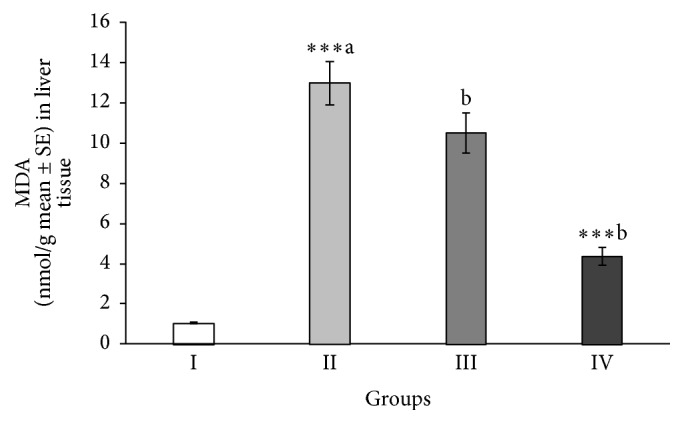
Effect of* A. biebersteinii* oil on the levels of MDA in the liver tissue of the rats. Group I: control; Group II: CCl_4_; Group III: ABEO (0.2 mL/kg + CCl_4_); Group IV: silymarin (10 mg/kg + CCl_4_); (a) as compared with normal group, (b) as compared with CCl_4_ group.

As presented in Figure 1, the concentration of hepatic MDA, a final product of lipid peroxidation, in the rats treated by CCl_4_ that did not receive ABEO was significantly (*p* < 0.001) high compared to the normal control rats. Interestingly, the rats pretreated with ABEO (0.2 mL/kg) caused a significant decrease in the level of MDA (Figure 1). Also, similar results were obtained in rats treated with silymarin.

### 3.6. Effect of ABEO on Hepatic NP-SH

**Figure 2:**
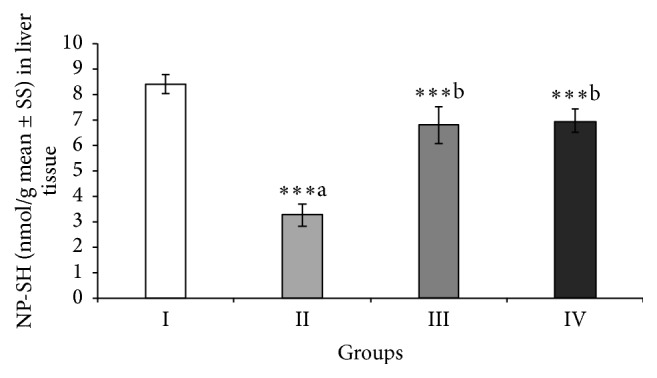
Effect of* A. biebersteinii* on the levels of nonprotein sulfhydryl (NP-SH) in the liver tissue of the rats. Group I: control; Group II: CCl_4_; Group III: ABEO (0.2 mL/kg + CCl_4_); Group IV: silymarin (10 mg/kg + CCl_4_); (a) as compared with normal group, (b) as compared with CCl_4_ group.

As shown in Figure 2, the decreased level of NP-SH in the liver tissues of animals treated with CCl_4_ was significantly (*p* < 0.001) lifted by ABEO. The same biological effect was noticed in rats treated with silymarin.

### 3.7. Effect of ABEO on Hepatic TP

**Figure 3:**
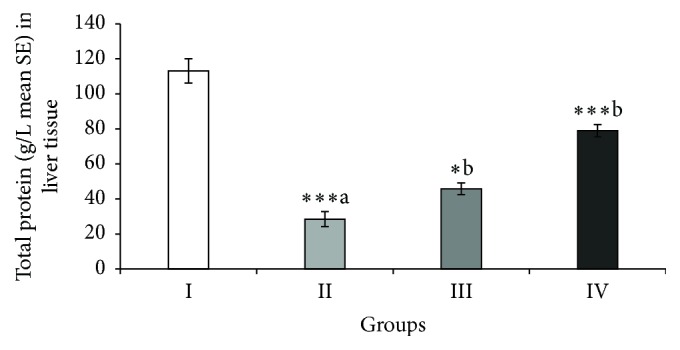
Effect of* A. biebersteinii* on the levels of total protein (TP) in the liver tissue of the rats. Group I: control; Group II: CCl_4_; Group III: ABEO (0.2 mL/kg + CCl_4_); Group IV: silymarin (10 mg/kg + CCl_4_); (a) as compared with normal group, (b) as compared with CCl_4_ group.

CCl_4_ caused significant decrease of the total protein level in Group II (Figure 3). ABEO elevated significantly the protein level in the liver tissue. Silymarin as reference drug produced a significant increase in total protein concentration.

### 3.8. Effect of ABEO on Histopathological Evaluation

**Figure 4:**
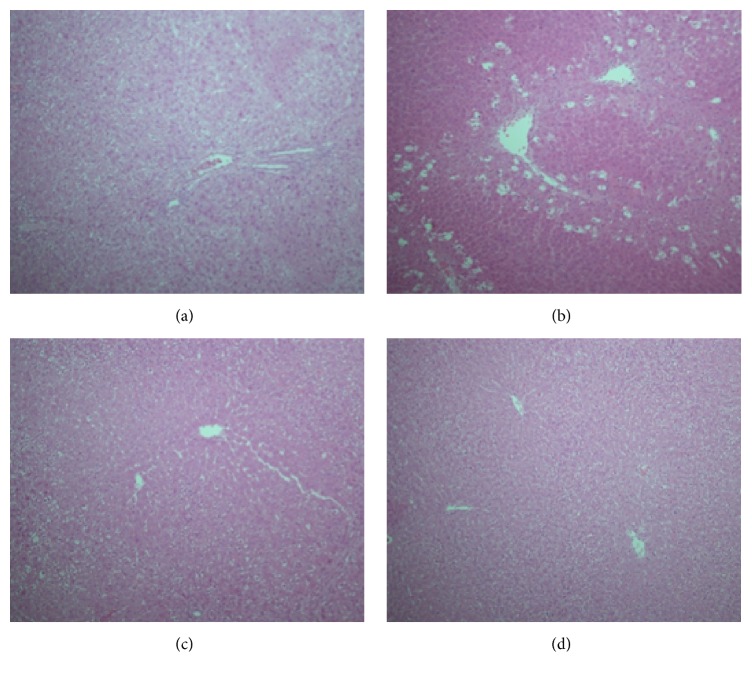
Light micrographs showing the effect of essential oil of* Achillea biebersteinii* (ABEO) on CCl_4_-induced hepatotoxicity in rats. (a) Normal hepatocytes. (b) CCl_4_-induced severe necrosis and inflammation. (c) Pretreatment of rats with ABEO. (d) Pretreatment of rats with silymarin.

As demonstrated in Figure 4, the histopathological investigations reinforced the data of the biochemical analysis. The micrographs of the liver tissues exhibited serious necrosis and focal hepatic cellular decay of the lobules in rats treated by CCl_4_ comparing with normal control rates. Basically normal hepatocytes and central vein were observed in the rats treated with ABEO (0.2 mL/kg). In addition, liver tissues of rats treated with silymarin exhibited normal central vein, sinusoids hepatocytes (Figure 4).

### 3.9. Antioxidant Activity of ABEO

As shown in Table 4, ABEO demonstrated a moderate radical scavenging activity particularly at the highest levels of 500 and 1000 *μ*g/mL (42 and 58%) whereas ascorbic acid exhibited a strong activity (92 and 94%). Furthermore, Table 4 showed the antioxidant activity, dependent on the *β*-carotene bleaching rate of the ABEO. The observed antioxidant activity of ABEO was moderate (53%) in comparison to that of rutin (91%).

## 4. Discussion

In this research, we investigated the chemical composition and* in vivo* hepatoprotective and* in vitro* antioxidant activity of the volatile oil of* Achillea biebersteinii* (ABEO). To the best of our knowledge this study represents the first investigation of ABEO potential to ameliorate hepato-toxicity induced by CCl_4_. Our findings that suggest *α*-terpinene (29%) and* p*-cymene (23%) as main constituents of the essential oil (ABEO) in our study are in agreement with an earlier study on* A*.* biebersteinii* collected from East Azerbaijan, Iran, where *α*-terpinene (41%) and* p*-cymene (13%) were the major components [24]. Other reports on the chemical composition of* A. biebersteinii* essential oil from Turkey and Iran showed a main chemotype which is characterized by the presence of 1,8-cineole (9–37%) and camphor (16–30%) as major components [4, 25, 26]. Moreover, the GC-MS analysis of the oil of* A. biebersteinii* collected from Sivas in Turkey has resulted in the identification of piperitone (35%) and eucalyptol (13%) as the main components [6]. Sökmen et al. [6] attributed the richness of these monoterpenes to the collection of the plant material during flowering period. However, we assume that the variation in the chemical composition could be attributed to several factors including the geographical origin, environmental factors in Saudi Arabia, genetic type, and development stage. Previous biological and pharmacological studies on* A. biebersteinii* demonstrated that the essential oil as well as the crude extract have antibacterial, antifungal, insecticidal, and antioxidant activities [4, 6, 26]. Moreover, Akkol et al. [27] reported remarkable wound healing activity. Recent studies on* A. biebersteinii* recorded antileishmanial as well as protective and therapeutic effects against gastric ulcer [28, 29].

In an endeavor to evaluate the hepatoprotective effect of ABEO, CCl_4_-induced hepatotoxicity model was undertaken in this investigation. CCl_4_ is a well-known and commonly used chemical to promote liver injury. The mechanism of CCl_4_-induced hepatotoxicity is basically mediated through certain free radical reactions [30]. The toxicity with CCl_4_ exists in its metabolic biotransformation by cytochrome P450 system in liver tissues to give two reactive free radicals, namely, trichloromethyl (CCl_3_^∙^) and trichloromethylperoxy (CCl_3_OO^∙^), which initiate lipid peroxidation process and decrease activities of antioxidant enzyme levels [31–33]. In the current study, CCl_4_ caused a significant increase in the serum marker enzymes (GOT, GPT, GGT, and ALP) and bilirubin indicating a wide disturbance in structure and function of liver cells. The results showed that administration of ABEO as well as silymarin significantly restored that pathological increase of these enzymes and indicate a hepatoprotective effect against CCl_4_-induced liver damage. Our findings are in agreement with the result demonstrated by Dadkhah et al. [34] for the hepatoprotective effect of the essential oil of* A. wilhelmsii*, which showed a modulation of the increased enzyme markers. In addition, our results were consistent with the reported results on* A. millefolium* extract by Yaeesh et al. [35] who demonstrated a decrease of the enzyme levels, for example, ALT and AST, in treated animal groups compared with the control suggesting a hepatoprotective activity for* A. millefolium*. Surprisingly our findings were unlike the results reported on the ethanol extract of* A. biebersteinii* [36]. On the contrary, the ethanol extract of* A. biebersteinii* induced an increase in AST and ALT in a dose-dependent manner [36].

In addition to that, our results showed a noticeable increase in malondialdehyde (MDA) level in liver tissues by CCl_4_ indicating enhancement of lipid peroxidation (LPO) which is an essential pathogenic cause for tissue damage [37]. MDA is an end product of lipid peroxidation in liver tissue and is used as a marker of LPO, which takes place in liver toxicity due to the formation of reactive oxygen species (ROS) [37, 38]. A significant decrease of MDA level was observed in rats, which were treated with ABEO and silymarin. Probably ABEO protects the liver cells by prevention of the generation of free radical species and so the decreasing of lipid peroxidation mediated by CCl_4_ [39].

Another indicator of hepatotoxicity is the consumption of nonprotein sulfhydryl (NP-SH) content of the liver tissues which could induce further damage and dysfunction of liver [38]. ABEO as well as silymarin increased significantly the level of NP-SH in liver tissues indicating a hepatoprotective effect in the treated animals. Moreover, the depletion of total protein (TP) has been shown to be an indicator for oxidative stress and liver toxicity. CCl_4_ significantly induced a decrease of total protein (TP) in CCl_4_-treated animals indicating hepatotoxicity. Restoring the levels of TP by ABEO and silymarin denotes a reduction of oxidative stress and thus hepatoprotective effect. Beside the hepatoprotective activity, ABEO showed a relatively moderate* in vitro* DPPH radical scavenging and antioxidant activity. Our results are in agreement with earlier data published by Sökmen et al. [6], who indicated antioxidant activity for the oil. In general, both effects (hepatoprotective and antioxidative effects) might be in relation to each other.

## 5. Conclusions

In conclusion, by evaluation of the* in vivo* hepatoprotective and* in vitro* antioxidant effects of* A. biebersteinii* essential oil (ABEO), it has been found that ABEO showed significant protection against CCl_4_-induced liver injury, as well as dose-dependent antioxidant activity. The present results confirm a beneficial association between the hepatoprotective effectiveness and the antioxidant activity of the ABEO and support its use in liver disorders by folk medicine practitioners.

## Figures and Tables

**Table 1:** Chemical composition of the essential oil of *A. biebersteinii*.

Number	Compounds	RI	%	Identification
1	*α*-Pinene	1032	1.4	1,2,3
2	*α*-Thujene	1035	0.1	1,2
3	Camphene	1076	0.1	1,2,3
4	*β*-Pinene	1118	0.2	1,2
5	Sabinene	1132	0.2	1,2
6	*α*-Phellandrene	1176	0.5	1,2
7	*α*-*Terpinene*	1188	*29.2*	1,2
8	Dehydro-1,8-cineole	1195	0.1	1,2,3
9	Limonene	1203	0.1	1,2
10	1,8-Cineole	1213	4.3	1,2,3
11	*γ*-Terpinene	1255	0.9	1,2,3
12	*p-Cymene*	1280	*22.9*	1,2,3
13	Terpinolene	1290	0.1	1,2,3
14	1,3,8*-p-*Menthatriene	1408	0.1	1,2
15	(*E*)-2-Hexenol	1412	0.1	1,2,3
16	*p*-Cymenene	1452	0.5	1,2,3
17	Camphor	1532	0.9	1,2,3
18	Linalool	1553	0.5	1,2,3
19	*trans*-*p*-Menth-2-en-1-ol	1571	3.9	1,2,3
20	Terpinen-4-ol	1611	4.7	1,2,3
21	*cis-p*-Menth-2-en-1-ol	1638	2.9	1,2
22	*trans*-Pinocarveol	1664	0.8	1,2
23	*trans*-Piperitol	1689	1.1	1,2
24	*α*-Terpineol	1706	1.1	1,2,3
25	Borneol	1719	tr	1,2,3
26	Germacrene D	1726	0.3	1,2,3
27	*trans*-Piperitone oxide	1755	2.5	1,2
28	*cis*-Piperitol	1758	1.7	1,2
29	Cuminaldehyde	1802	0.4	1,2
30	*p*-Mentha-1,3-dien-7-al	1811	0.3	1,2
31	*p*-Cymen-8-ol	1864	1.0	1,2
32	Ascaridole	1889	3.1	1,2
33	Eugenol	2185	0.3	1,2,3
34	*γ*-Eudesmol	2186	1.1	1,2
35	Thymol	2198	0.9	1,2,3
36	Eromoligenol	2205	tr	1,2
37	Isocarvacrol	2221	0.3	1,2
38	Carvacrol	2239	2.1	1,2,3
39	*β*-Eudesmol	2257	0.9	1,2,3
40	15-Hexadecanolide	2260	0.4	1,2
41	1,4-Dimethyl azulene	2291	tr	1,2
42	(*Z*)-methyl jasmonate	2365	tr	1,2
43	Chamazulene	2430	tr	1,2
44	*γ*-Costol	2533	tr	1,2
	Monoterpene hydrocarbons		56.3	
	Oxygenated monoterpenes		31.9	
	Sesquiterpene hydrocarbons		0.3	
	Oxygenated sesquiterpenes		2.0	
	Other compounds		1.8	
	Total		92.0	

RI: retention indices relative to C8–C30 n-alkanes on the Innowax FSC column; 1: retention index; 2: mass spectrum; 3: spiking with authentic compound.

**Table 2:** Effect of *A. biebersteinii* essential oil (ABEO) on induced hepatotoxicity-related parameters.

Treatment group (*n* = 6)	SGOT (U/L)	SGPT (U/L)	GGT (U/L)	ALP (U/L)	Bilirubin (mg/dL)
Normal control	79.66 ± 4.40	42.83 ± 2.77	7.58 ± 0.27	346.33 ± 9.40	0.57 ± 0.01
CCl_4_ only	319.33 ± 10.85^*∗∗∗*a^	270.16 ± 21.23^*∗∗∗*a^	19.15 ± 1.19^*∗∗∗*a^	624.83 ± 11.76^*∗∗∗*a^	2.84 ± 0.17^*∗∗∗*a^
ABEO (0.2 mL/kg + CCl_4_)	217.22 ± 9.75^*∗∗∗*^	176.83 ± 8.13^*∗∗*^	11.08 ± 0.57^*∗∗∗*^	535.33 ± 12.36^*∗∗*^	1.80 ± 0.07^*∗∗∗*^
Silymarin (10 mg/kg + CCl_4_)	147.5 ± 10.85^*∗∗∗*b^	118.18 ± 7.48^*∗∗∗*b^	9.06 ± 0.35^*∗∗∗*b^	413.81 ± 13.60^*∗∗*b^	1.29 ± 0.08^*∗∗∗*b^

All values represent mean ± SEM. ^*∗*^ *p* < 0.05; ^*∗∗*^ *p* < 0.01; ^*∗∗∗*^ *p* < 0.001; ANOVA, followed by Dunnett's multiple comparison test. ^a^As compared with normal group. ^b^As compared with CCl_4_ only group.

**Table 3:** Effect of *A. biebersteinii* essential oil (ABEO) on CCl_4_-induced lipid profile in rats.

Treatment group (*n* = 6)	Cholesterol (mg/dL)	Triglycerides (mg/dL)	HDL (mg/dL)	LDL (mg/dL)	VLDL (mg/dL)
Normal control	95.01 ± 4.11	52.83 ± 2.52	54.63 ± 2.26	73.80 ± 2.80	24.01 ± 1.14
CCl_4_ only	148.5 ± 5.09^*∗∗∗*a^	144.5 ± 4.70^*∗∗∗*a^	19.45 ± 1.38^*∗∗∗*a^	21.26 ± 1.33^*∗∗∗*a^	65.68 ± 2.14^*∗∗∗*a^
ABEO (0.2 mL/kg + CCl_4_)	117.16 ± 4.06^*∗∗∗*b^	116.0 ± 2.58^*∗∗∗*b^	32.66 ± 2.81^*∗∗*b^	33.19 ± 3.60^*∗*b^	52.72 ± 1.17^*∗∗∗*b^
Silymarin (10 mg/kg) + CCl_4_	108.93 ± 5.18^*∗∗∗*b^	61.50 ± 11.07^*∗∗∗*b^	40.73 ± 3.27^*∗∗*b^	62.49 ± 5.12^*∗∗*b^	29.75 ± 5.03^*∗∗∗*b^

All values represent mean ± SEM. ^*∗*^ *p* < 0.05; ^*∗∗*^ *p* < 0.01; ^*∗∗∗*^ *p* < 0.001; ANOVA, followed by Dunnett's multiple comparison test. ^a^As compared with normal group. ^b^As compared with CCl_4_ only group.

**Table 4:** Free radical scavenging activity and antioxidant activity of the *A. biebersteinii* essential oil (ABEO).

Plant species	Radical scavenging activity (%)					Total antioxidant activity (%)
	10	50	100 (*µ*g/mL)	500	1000	1000 (*µ*g/mL)
ABEO	3.5	15.9	23.1	42.2	58.5	53.1
Ascorbic acid	16.9	68.5	85.8	91.4	94.2	—
Rutin	—	—	—	—	—	91.2
